# Efficacy of LVIS vs. Enterprise Stent for Endovascular Treatment of Medium-Sized Intracranial Aneurysms: A Hemodynamic Comparison Study

**DOI:** 10.3389/fneur.2019.00522

**Published:** 2019-05-28

**Authors:** Wenqiang Li, Yang Wang, Yisen Zhang, Kun Wang, Ying Zhang, Zhongbin Tian, Xinjian Yang, Jian Liu

**Affiliations:** ^1^Department of Interventional Neuroradiology, Beijing Neurosurgical Institute and Beijing Tiantan Hospital, Capital Medical University, Beijing, China; ^2^Department of Neurosurgery, The First Affiliated Hospital of Nanchang University, Nanchang University, Nanchang, China

**Keywords:** intracranial aneurysms, hemodynamics, stent, endovascular treatment, computational fluid dynamics

## Abstract

**Background:** We conducted a computational fluid dynamics (CFD) study and compared the treatment of medium-sized intracranial aneurysms with LVIS and Enterprise stent-assisted coil embolization (SACE) to determine the effects of hemodynamic changes caused by different stent and coil packing densities (PDs) in endovascular treatment.

**Methods:** We enrolled 87 consecutive patients, with 87 medium-sized intracranial aneurysms (≥7, ≤ 12 mm), who underwent LVIS or Enterprise SACE. Aneurysms treated with LVIS SACE were allocated to the LVIS group, and the remainder were allocated to the Enterprise group. CFD were performed to assess hemodynamic alterations between before treatment, after stent deployment, and after SACE.

**Results:** One aneurysm recanalized in the LVIS group (*n* = 42), and five recanalized in the Enterprise group (*n* = 45) (recanalization rate: 2.4 vs. 11.1%, respectively; *P* = 0.108). Higher complete obliteration rate (*P* = 0.069) was found in the LVIS group. Velocity at the neck plane showed a greater reduction ratio than velocity and WSS of the aneurysm in both groups after stent deployment, while velocity and WSS of the aneurysm showed a greater reduction ratio after coil placement. Further, there was a greater reduction in velocity at the neck plane (59.52 vs. 39.81%), aneurysmal velocity (88.46 vs. 69.45%), and wall shear stress (WSS) (85.45 vs. 69.49%) on the aneurysm in the LVIS group (*P* < 0.001 for all). Specifically, the reduction ratio of velocity at the neck plane showed significant difference between the groups in the multivariate analysis (*P* = 0.013).

**Conclusions :** LVIS SACE showed a lower recanalization for endovascular treatment of medium-sized intracranial aneurysms, and the greater hemodynamic alterations might be the key factors.

## Introduction

Stent-assisted coil embolization (SACE) is a popular treatment strategy for wide-necked intracranial aneurysms (IAs) and is associated with a higher occlusion rate and lower recurrence rate (1–5). From laser cut stents (e. g., Enterprise stent) to braided stents (e. g., LVIS) and now the flow diverter (e. g., Pipeline embolization device), the design and concept of stents used for the treatment of IAs are continuously developing. The efficacy of endovascular treatment with these devices was also reported to increase with their development. For example, the occlusion rate of IAs treated by flow diverter is >90% (6). However, the indication of the flow diverter is strict, and it is commonly used in large or giant aneurysms. In recent years, a novel, self-expandable braided stent (LVIS) has become widely used, which has a smaller cell structure and a higher metal coverage than the Enterprise stent (7–9). The angiographic outcome of IAs treated with LVIS was reported to be favorable (9–13). For example, in a previous study from our center, the recanalization rate of aneurysms with an LVIS stent was lower than aneurysms treated with the Enterprise stent, while the rate of progressive thrombosis occlusion was significantly higher in the LVIS group (10). However, the underlying mechanisms of action remain controversial.

Hemodynamic alterations induced by stents were reported to be involved in aneurysm outcomes. Computational fluid dynamics (CFD) is a valuable tool for evaluating the hemodynamic effects of aneurysm recurrence (14–16). However, the hemodynamic effects of LVIS in aneurysm outcomes remain unknown, and to our knowledge, there are no reported hemodynamic comparison studies based on a series of clinical cases. Thus, in the present study, we retrospectively collected data from patients with IAs who were treated with LVIS and Enterprise stents in our center. For large or giant aneurysms, the flow diverter was reported to be a better treatment method (17, 18). However, in those studies, the indication of the flow diverter was strict, and complex aneurysm (large, giant, and fusiform aneurysms) was the main indication. For medium-sized IAs (≥7, ≤ 12 mm) (19), the traditional stent-assisted technique may be more appropriate for its low metal coverage and dense packing. By contrast, the Enterprise and LVIS stents are the two most commonly used intracranial stents that have different structure designs. We hypothesized that differences in the hemodynamic effects of these stents may underlie the different outcomes of aneurysms treated with these two devices.

## Materials and Methods

### Patient Selection

The study was approved by the ethics committee of Beijing Tiantan hospital. Consent was obtained from the patients or their closest relatives before the study. We retrospectively reviewed the medical records and image data in our aneurysm database. A series of patients were reviewed and collected from February 2016 to January 2017. Patients were screened retrospectively based on the following inclusion criteria: (1) diagnosis of medium-sized aneurysms by angiography (size range from 7 to 12 mm) and treated with LVIS (MicroVention, Tustin, CA, USA) or Enterprise SACE (Cordis Neurovascular, Miami, FL, USA), (2) available follow-up angiography performed to determine whether the aneurysm had recanalized, and (3) sufficient resolution of three-dimensional (3D) digital subtraction angiography images for CFD simulation. Two experienced neurointerventionalists (5 years of experience in endovascular treatment) who were independent of the study evaluated the angiography results and radiographic images, and disagreements were resolved by a third neurointerventionalist (10 years of experience in endovascular treatment). According to the Raymond–Roy classification system (20), the angiographic results of aneurysm embolization were quantified (class 1, complete obliteration; class 2, a residual neck; class 3, a residual aneurysm). Recanalization was defined as any further contrast filling of the aneurysm sac observed on follow-up angiography compared with the initial treatment results (14). Aneurysms treated with the LVIS stent were allocated to the LVIS group, while aneurysms treated with the Enterprise stent were allocated to the Enterprise group. For all included cases, clinical data [age, sex, hypertension, cigarette smoking, multiple aneurysm, aneurysm location, packing density (PD), follow-up interval, complication rates, and retreatment rates], and morphological data (aneurysm size, neck size, and aspect ratio) were collected from medical records and imaging studies.

### Computational Modeling, Hemodynamic Simulations, and Hemodynamic Analysis

Patient-specific 3D-digital subtraction angiography data of all aneurysms were obtained before treatment. Using Geomagic Studio version 12.0 software (Geomagic, Research Triangle Park, NC, USA), the 3D geometry surface was displayed, segmented, and smoothed. The geometries were saved in standard tessellation language format. A novel virtual stenting technique (21, 22) and porous media method (23) were used to simulate the *in vivo* stent and coil mass in the aneurysm dome region. The virtual stenting workflow was as follows: (1) preprocessing: isolated the parent vessel from the aneurysm and trimmed down the stent deployment region; (2) simplex mesh expansion: the centerline was obtained within the parent vessel to undergo radial expansion to initialize a simplex mesh, and the expansion was stopped when the initialized simplex mesh had good apposition with the parent–vessel wall; (3) post-processing: the LVIS and Enterprise stent pattern was mapped to the simplex mesh, and the wires were swept into the 3D structures using CAD software (Creo Parametric 2.0; PTC Needham, MA, USA) (22). The aneurysmal sac with coils was modeled as a porous medium as described by Mitsos et al. (23). The virtual stent was merged with the aneurysm geometry using ICEM CFD software (ANSYS, Inc., Canonsburg, PA, USA) to create >1,000,000 finite-volume tetrahedral elements. The maximum element size and the element size on stents were set at 0.2 mm and one-third of the width of the strut of these stents approximately. ANSYS CFX 14.0 software (ANSYS, Inc.,) was used to simulate the hemodynamics of the aneurysm after meshing. We treated blood as a Newtonian fluid. The blood vessel wall was assumed to be rigid with no-slip boundary conditions. The density was specified as ρ = 1,060 kg/m^3^, and the dynamic viscosity of blood was μ = 0.004 Pa·s. The governing equations underlie the calculation based on the Navier–Stokes formula, with the assumption of homogeneous, laminar, incompressible blood flow. The inflow boundary condition was obtained using transcranial Doppler imaging as a representative pulsatile period velocity profile. The outlet pressure conditions at outlet arteries in our study were imposed to p = 0 Pa. The flow waveforms were scaled to achieve a mean inlet wall shear stress (WSS) of 15 dynes/cm under pulsatile conditions (22). To confirm the stability, we selected the third cardiac cycle of three cardiac cycle simulations as output for the final analyses.

We then post-processed and visualized the results of these simulations using the ANSYS CFD-Post. The hemodynamic results at peak systole were carefully examined. In our clinical practice, the jailing technique was used in the present cases. We first deployed a stent to jail the microcatheter and then performed coil embolization. Thus, we created a group of three models (before treatment, after stent deployment, and after SACE), compared the flow alterations during the entire procedure between the three models, and estimated the hemodynamic effects of stenting and coiling. All results were collected before stent placement, after stent placement, and after SACE. The hemodynamic parameters were collected at systolic peak. The average flow velocity at the aneurysm neck plane was calculated, and the aneurysm neck plane was created at the location where the aneurysm sac pouched outward from the parent artery. Moreover, the average flow velocity inside the aneurysm and the average WSS on the whole aneurysm wall were also calculated. We defined the reduction ratios of the parameter as (pretreatment parameter—post-treatment parameter)/pretreatment parameter. For hemodynamic parameters, the reduction ratios of the flow velocity at the aneurysm neck plane, the flow velocity in the aneurysm, and WSS on the aneurysm were analyzed. Hemodynamic alterations by stent deployment were defined as the reduction ratios between before and after stent placement, hemodynamic alterations by coil embolization were the reduction ratios between after stent placement and after SACE, and total hemodynamic alterations were the reduction ratios between before and after SACE.

### Statistical Analysis

For qualitative data, the χ2 test or the Fisher's exact test was used to compare the differences between the LVIS and Enterprise groups. For quantitative data, the Mann–Whitney *U*-test was used to compare between the two groups. The factors with a *P* < 0.2 in univariate analysis were entered into a multivariate logistic regression analysis to assess the independent factors related to treatment. The OR with 95% CIs was calculated between the LVIS and Enterprise group. Statistical analyses were performed using statistical software (SPSS version 21.0; SPSS Inc., Chicago, IL, USA). The level of statistical significance was established at *P* < 0.05.

## Results

After review, 87 patients with 87 medium-sized IAs were treated with LVIS SACE or Enterprise SACE in our study. Forty-two aneurysms were treated with LVIS and 45 with the Enterprise stent. One aneurysm was recanalized in the LVIS group, while five aneurysms were recanalized in the Enterprise group. Aneurysms in the LVIS group showed a lower recanalization rate (2.4 vs. 11.1%, respectively; *P* = 0.108; Table 1).

**Table 1:** Patient demographics and aneurysm morphology in patients with Enterprise stent-assisted coil embolization (SACE) and LVIS SACE.

	**Enterprise group (*n* = 45)**	**LVIS group (*n* = 42)**	***P*-value**
Age, y	55.38 ± 9.61	53.60 ± 8.55	0.417
Female sex, *n* (%)	35 (77.8)	30 (71.4)	0.496
Hypertension (HTN), *n* (%)	12 (22.7)	8 (19.0)	0.675
Cigarette smoking, *n* (%)	6 (13.3)	5 (11.9)	0.841
Drinking, *n* (%)	5 (11.1)	3 (7.1)	0.522
Ruptured aneurysms, *n* (%)	9 (20.0)	5 (11.9)	0.305
Multiple aneurysms, *n* (%)	11 (24.4)	7 (16.7)	0.371
Anterior circulation, *n* (%)	42 (93.3)	39 (92.9)	0.930
Aneurysm size, mm	8.95 ± 1.82	8.81 ± 1.89	0.718
Aneurysm neck, mm	6.25 ± 1.78	5.87 ± 1.40	0.273
Aspect ratio (AR)	1.50 ± 0.38	1.54 ± 0.29	0.579
Packing density (%)	25.58 ± 6.44	28.34 ± 7.01	0.146
Initial angiographic result			0.069
Complete obliteration	20 (44.4)	29 (69.0)	
Residual neck	21 (46.7)	11 (26.2)	
Residual aneurysm	4 (8.9)	2 (4.8)	
Follow-up interval, mo	11.82 ± 9.26	9.10 ± 4.01	0.932
Thromboembolic complications, *n* (%)	4 (8.9)	5 (11.9)	0.644
Hemorrhagic complications, *n* (%)	1 (2.2)	1 (2.4)	0.961
Recanalization, *n* (%)	5 (11.1)	1 (2.4)	0.108
Retreatment, *n* (%)	3 (6.7)	1 (2.4)	0.340

*Continuous variables are expressed as mean ± standard deviation. Categorical variables are expressed as n (%)*.

### Clinical and Morphological Factors

Patient demographics and aneurysm morphology of the LVIS and Enterprise groups are shown in Table 1. There were no significant differences in any factors between the two groups. However, patients tended to be younger with less women in the LVIS group. The mean maximum size and neck of the aneurysm in the Enterprise group was also slightly larger than that in the LVIS group, while the AR was slightly smaller. There was a trend toward a higher complete obliteration rate in the LVIS group compared with the Enterprise group (69.0 vs. 44.4%, respectively; *P* = 0.069). The PD of aneurysms in the LVIS group was higher (28.34 ± 7.01 vs. 25.58 ± 6.44%, respectively). Further, the follow-up interval time was longer in the Enterprise group. There were no significant differences in the rate of complications between the two groups: thromboembolic complications occurred in five patients (11.9%) in the LVIS group and in four patients (8.9%) in the Enterprise group (*P* = 0.644). Regarding hemorrhagic complications, intraoperative rupture occurred in one patient in the LVIS group and in one patient in the Enterprise group (*P* = 0.961). The retreatment rate also showed no significant differences between the two groups (*P* = 0.340).

### Hemodynamic Alterations by Staged SACE

The alterations of hemodynamic parameters between the LVIS and Enterprise group by staged SACE are shown in Table 2.

**Table 2:** Univariate analysis results for hemodynamic parameters between the two groups.

**Parameters**	**Enterprise group (*n* = 45), Reduction rate (%)**	**LVIS group (*n* = 42), Reduction rate (%)**	***P*-value**
**VELOCITY ON THE NECK PLANE**			
Stent	26.70 ± 15.71	44.74 ± 18.14	< 0.001
Coil	13.91 ± 32.29	26.20 ± 31.38	0.185
Total	39.81 ± 17.76	59.52 ± 23.63	< 0.001
**VELOCITY ON THE ANEURYSM**			
Stent	22.59 ± 12.95	28.65 ± 14.84	0.021
Coil	61.18 ± 20.19	83.78 ± 17.66	< 0.001
Total	69.45 ± 17.78	88.46 ± 13.30	< 0.001
**WSS ON THE ANEURYSM**			
Stent	20.15 ± 12.26	23.33 ± 13.95	0.281
Coil	60.40 ± 20.36	81.54 ± 21.40	< 0.001
Total	69.49 ± 15.01	85.45 ± 18.12	< 0.001

*The Mann–Whitney u test was used, with P < 0.05 considered statistically significant. WSS, wall shear stress; Stent, hemodynamic alterations by stent deployment; Coil, hemodynamic alterations by coil placement; Total, total hemodynamic alterations after SACE*.

### Hemodynamic Alterations by Stent Deployment

For alterations in hemodynamic parameters by stent deployment, the hemodynamic parameters in both groups showed a greater reduction ratio for the velocity at the neck plane compared with the velocity and WSS of the aneurysm. However, in the LVIS group, the reduction ratio for the velocity at the neck plane (44.74 ± 18.14 vs. 26.70 ± 15.71, respectively; *P* < 0.001) and velocity of the aneurysm (28.65 ± 14.84 vs. 22.59 ± 12.95, respectively; *P* = 0.021) was significantly different than for the Enterprise group. The reduction of WSS on the aneurysm was also higher but showed no significance (*P* = 0.281).

### Hemodynamic Alterations by Coil Embolization

For the alterations of hemodynamic parameters by coil embolization after stent deployment, there were further reductions in the velocity at the neck plane, velocity in the aneurysm, and WSS on the aneurysm after coil placement. However, the velocity in the aneurysm and WSS on the aneurysm showed a greater reduction ratio compared with the velocity at the neck plane for both groups. Moreover, the velocity in the aneurysm (83.78 ± 17.66 vs. 61.18 ± 20.19, respectively; *P* < 0.001) and WSS at the aneurysm (28.65 ± 14.84 vs. 22.59 ± 12.95, respectively; *P* = 0.021) were significantly lower in the LVIS group compared with the Enterprise group, while there was no difference in velocity at the neck plane.

### Total Hemodynamic Alterations by SACE

**Figure 1:**
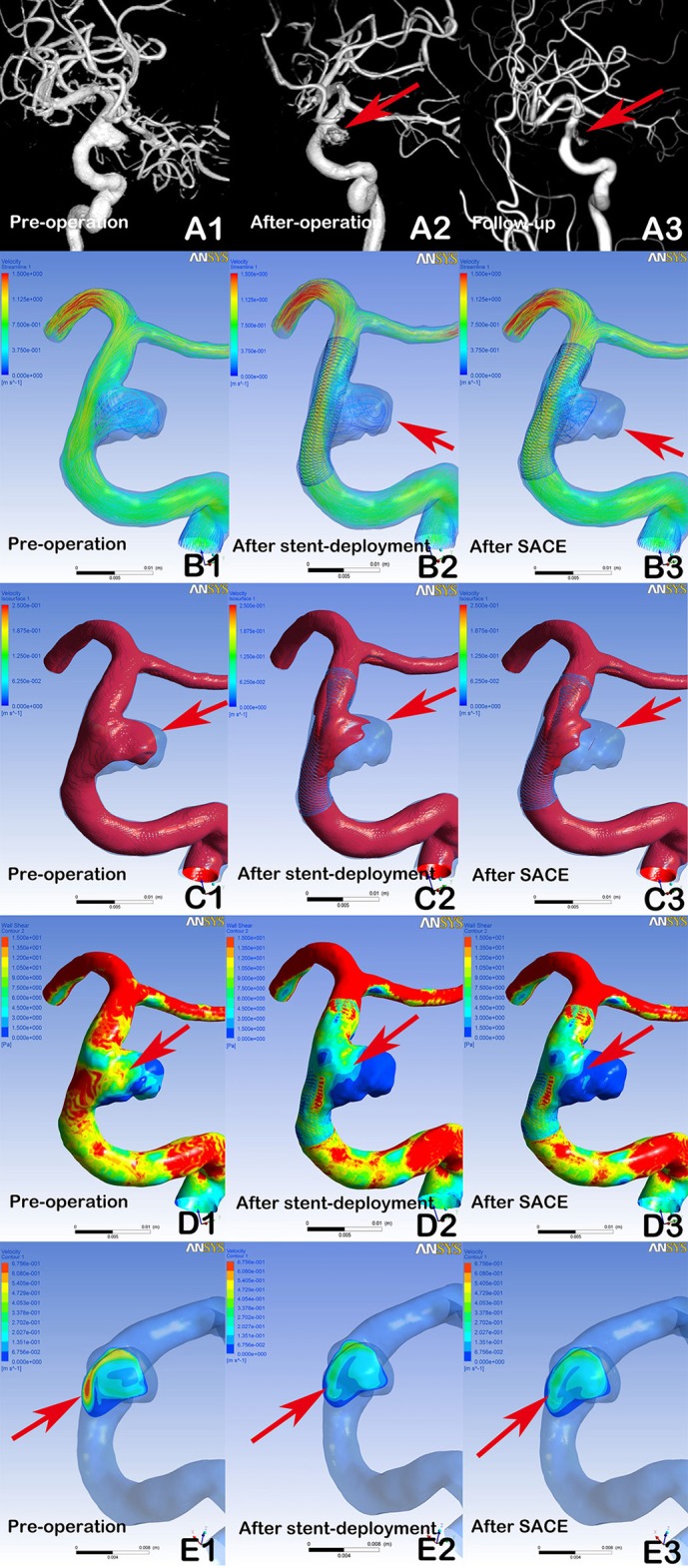
Digital subtraction angiography (DSA) showing a stable aneurysm (8.9 × 7.2 mm) with LVIS stent-assisted coil embolization (SACE) (A1, A2, A3, arrows). Compared with the preprocedural angiographic image (A1), the aneurysm showed residual flow in the post-procedural immediate angiographic image (A2, arrow). At 6 months of follow-up, DSA indicated that the aneurysm was stable (A3, arrow). In hemodynamic simulation, compared with preprocedural results (B1, C1, D1, E1), the velocity streamline was decreased (B2, arrow), the velocity in the aneurysm and wall shear stress (WSS) was decreased (C2 and D2, arrows), and the velocity of the aneurysm neck plane was markedly reduced (E2, arrow) after stent deployment. After coil placement, the streamline in the aneurysm and the velocity and WSS of aneurysm were further decreased (B3, C3, and D3), but with limited changes in velocity at the neck plane (E3).

**Figure 2:**
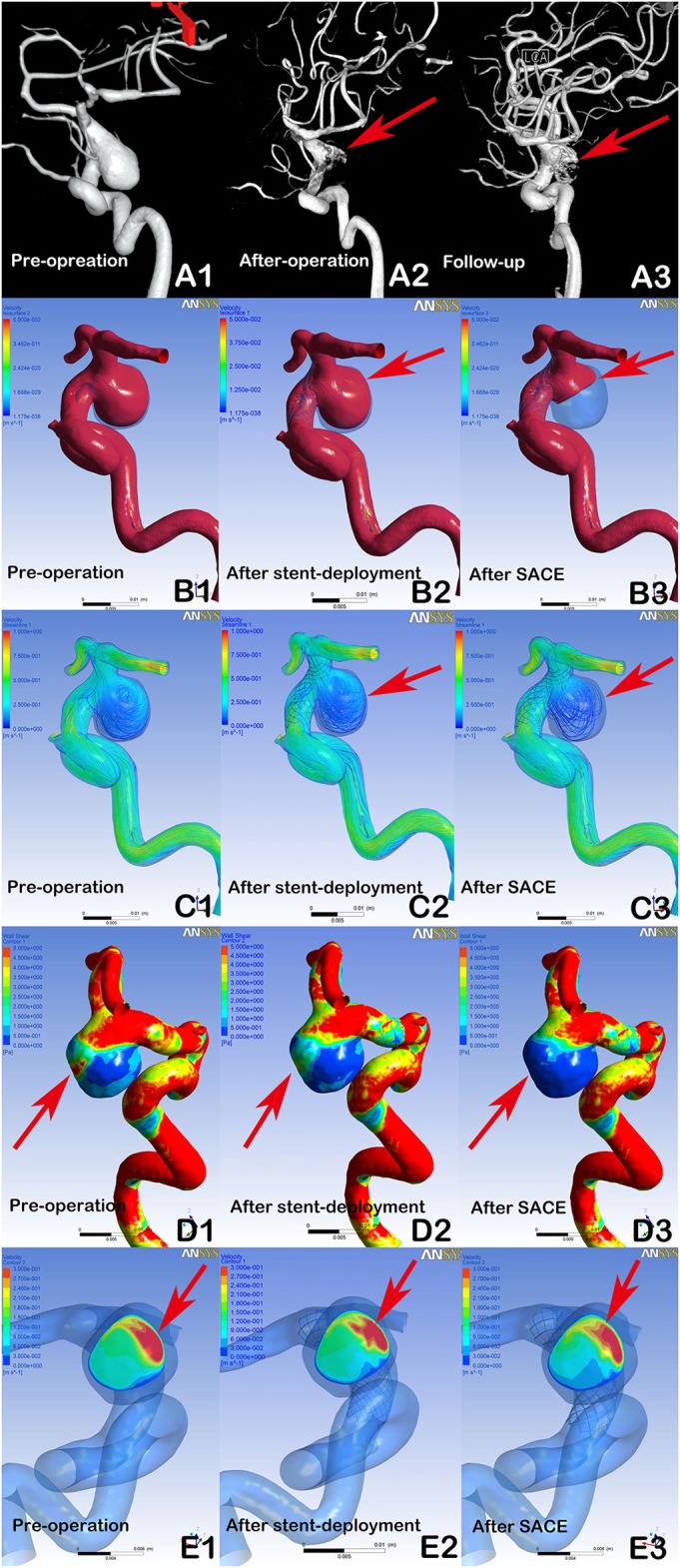
DSA showing a recanalized aneurysm (9.7 × 9.8 mm) with Enterprise SACE (A1, A2, A3, arrows). In post-procedural immediate angiographic images, the aneurysm showed a residual neck (A2, arrow). At 6 months of follow-up, DSA indicated that the aneurysm was recanalized (A3, arrow). In hemodynamic simulation, compared with preprocedural results (B1, C1, D1, E1), there were no marked changes in blood flow in the aneurysm after stent deployment (B2, C2, arrows). Further, there were limited changes in WSS on the aneurysm and velocity at the neck plane after stent deployment (D2, E2, arrows). With further coil placement (B3, C3, D3, E3), the velocity and WSS of the aneurysm had decreased. However, the blood flow velocity near the aneurysmal neck remained concentrated (B3, C3, arrows), the wall shear stress of the region near the aneurysmal neck remained high (D3, Arrows), and the impingement region at the remnant neck was consistent with the region where the recanalization occurred at follow-up (A3, E3, arrows).

For total hemodynamic alterations after SACE, there was a greater reduction ratio in the LVIS group for the various hemodynamic parameters (Table 2). Compared with the Enterprise group, cases with LVIS SACE showed a greater reduction in velocity at the neck plane (59.52 vs. 39.81%, respectively; *P* < 0.001), aneurysmal velocity (88.46 vs. 69.45%, respectively; *P* < 0.001), and WSS on the aneurysm (85.45 vs. 69.49%, respectively; *P* < 0.001). An unrecanalized example showed that blood flow in the aneurysms was remodeled and that the hemodynamics in aneurysm neck and aneurysm were obviously decreased after LVIS SACE (Figure 1). However, in the Enterprise group, the average velocity at the neck plane, velocity in the aneurysm, and WSS on the aneurysm decreased by 39.81, 69.45, and 69.49%, respectively, after treatment. A recanalized case with Enterprise SACE showed that the WSS and velocity of the aneurysm were decreased, while the velocity on the neck remained higher (Figure 2). Of all the significant factors in univariate analysis, only the reduction ratio of the velocity at the neck plane correlated significantly with treatment of different stent in multivariate logistic regression analysis (Table 3).

**Table 3:** Multivariate analysis between LVIS and Enterprise groups.

**Variables**	**OR# (95% CI)**	***P*-value**
Packing density	0.02 (0.00–760.22)	0.343
Initial angiographic result	0.19 (0.01–5.01)	0.320
Recanalization	0.12 (0.01–14.80)	0.386
Velocity on the neck plane (Stent)	0.80 (0.67–0.96)	0.014
Velocity on the neck plane (Coil)	0.88 (0.77–1.00)	0.054
Velocity on the neck plane (Total)	1.17 (0.96–1.43)	0.130
Velocity on the aneurysm (Stent)	1.02 (0.93–1.11)	0.704
Velocity on the aneurysm (Coil)	0.98 (0.73–1.30)	0.872
Velocity on the aneurysm (Total)	0.95 (0.65–1.38)	0.792
WSS on the aneurysm (Coil)	0.86 (0.73–1.01)	0.064
WSS on the aneurysm (Total)	1.17 (0.96–1.42)	0.120

*WSS, wall shear stress; Stent, hemodynamic alterations by stent deployment; Coil, hemodynamic alterations by coil placement; Total, total hemodynamic alterations after SACE*.

## Discussion

We performed a comparison study of LVIS and Enterprise SACE based on a series of clinical cases. The aneurysms with LVIS SACE showed a lower recanalization rate than those with Enterprise SACE. The velocity at the neck plane also showed a greater reduction after stent deployment compared with after coil placement, while the reduction ratio of the aneurysmal velocity and WSS was higher after coil placement. These findings suggest that coils mainly decrease the hemodynamics of the aneurysm dome, while stents mainly affect the velocity at the aneurysmal neck. Thus, the greater hemodynamic changes at the aneurysm neck and dome caused by the LVIS stent and high dense coils may play an important role in preventing aneurysm recanalization. In addition, the reduction ratio of the velocity at the neck plane was a significant independent factor between LVIS and Enterprise groups.

### Flow Remodeling Effect of Stents for Preventing Aneurysm Recanalization

SACE was developed from the concept that scaffolding in the parent artery of wide-necked aneurysms would prevent coil herniation (by stabilizing the coil) and aneurysm recurrence. Stent placement can also cause progressive endothelialization and reconstruction of the parent vessel. Moreover, an advantage of SACE is its potential for floreremodeling effects, with numerous studies showing that stents produce flroducesngtion effects to decrease the hemodynamics of the aneurysm (24–26). It is also well-established that flha diverters produce stronger fltrongersedson effects than for the Enterprise stent, with further hemodynamic reductions in the aneurysm (24–26). However, these studies mainly examined the hemodynamics of the Enterprise stent and flow-diverter device, while there is limited information on the effects of the LVIS stent on aneurysm hemodynamics. Nevertheless, in one study, the LVIS stent was reported to produce hemodynamic effects on cerebral aneurysms, with a single LVIS stent producing greater flow reductions than the two Enterprise stents, but less than for the Pipeline device (22). However, the findings in that study were not compared with clinical outcomes. In the present study, we selected two consecutive clinical cases to compare the hemodynamic alterations of aneurysms after SACE between patients treated with LVIS or the Enterprise stent. We found that greater reductions in velocity at the neck plane and in the aneurysm after LVIS stent deployment, and the reductions in velocity at the neck plane were the independent factors between the two groups. It was previously reported that a reduction in flow velocity at the neck plane was the most important factor for preventing aneurysm recanalization (27, 28). We found that the hemodynamic alterations in the LVIS group (including a significant reduction in velocity at the neck plane) were associated with a reduced recanalization risk. Thus, we speculate that the LVIS stent has a stronger flow diverter effect than the Enterprise stent, which may be important for preventing recanalization in middle IAs.

### Greater Hemodynamic Changes With Dense Coils may Relate to Lower Recanalization

A lower PD and complete obliteration rate were identified as significant predictors of aneurysm recurrence (16, 29). A higher PD and complete obliteration rate are achieved by providing a sufficient coil mass, which results in thrombus formation at the aneurysm and progressive endothelialization at the neck to prevent aneurysm recurrence. In the present study, higher PD and complete obliteration rate were found in the LVIS group, which may relate to the denser mesh used in the LVIS stent (a denser mesh stent can stabilize small coils and provide a higher coil PD). Further, there was a significant difference in the hemodynamic alterations at aneurysms caused by coil placement between the LVIS and Enterprise groups. The relationship between coil PD and hemodynamics alterations was also previously reported (30–32), with hemodynamics in the aneurysm reduced by coil insertion, and reduced intra-aneurysmal velocity and WSS associated with a higher PD. Based on these findings, we suggest that the use of dense coils following the LVIS stent provides greater hemodynamic effects on the aneurysm dome, which may also contribute to the lower recanalization rate in the LVIS group. A further hemodynamic reduction in the aneurysm dome when using dense coils may contribute to the further reduction in the recanalization rate for LVIS SACE.

Large or giant aneurysms may exhibit more complex flow patterns caused by pulsatile flow than that for small aneurysms, and require stronger flow remodeling effects to promote thrombosis formation (33). Thus, the flow diverter may be the best choice. However, for medium-sized aneurysms, the higher density mesh of the LVIS stent compared with the Enterprise stent can increase the coil density in the aneurysm dome to further decrease hemodynamics. Moreover, the higher metal coverage of the LVIS stent produces a stronger flow diverter effect to reduce flow velocity at aneurysm neck. Thus, the combined hemodynamic actions of LVIS stents and dense coils may be important for preventing aneurysm recanalization.

## Limitations

There are a number of limitations in the present study. First, the small sample size and mid-term follow-up may have influenced our findings, and a prospective study with a larger sample size should be performed for validation. Second, as for most CFD studies using vessel and aneurysm blood flow simulations, there are several assumptions (e.g., a rigid wall, laminar flow, general boundary conditions, and Newtonian blood) that may affect the hemodynamic results. Finally, the mechanisms of action of aneurysm occlusion cannot be explained by hemodynamics alone, with other factors having potential to influence outcomes, including thrombus formation, branching anatomy, branches arising from the aneurysm, the metal coverage of the stent, and luminal geometry changes.

## Conclusion

In the LVIS group, the greater reduction in velocity at the neck plane might be caused by a higher metal coverage of LVIS stent, and the greater reduction in velocity and WSS at the aneurysm might be caused by a higher PD with LVIS stent. The greater hemodynamic alterations may be key factors associated with the lower recanalization in medium-sized aneurysms treated with LVIS SACE.

## Data Availability

All datasets generated for this study are included in the manuscript and/or the supplementary files.

## Author Contributions

WL contributed to the preparation of the manuscript and data collection. JL contributed to the revision of the manuscript. YW, WL, YSZ, KW, ZT, and YZ contributed to data analysis and interpretation. JL and XY contributed to the experimental design and manuscript revision.

### Conflict of Interest Statement

The authors declare that the research was conducted in the absence of any commercial or financial relationships that could be construed as a potential conflict of interest.

## References

[1] Piotin M Blanc R Spelle L Mounayer C Piantino R Schmidt PJ. Stent-assisted coiling of intracranial aneurysms: clinical and angiographic results in 216 consecutive aneurysms. Stroke. (2010) 41:110–5. 10.1161/STROKEAHA.109.558114 19959540

[2] Lawson MF Newman WC Chi YY Mocco JD Hoh BL. Stent-associated flow remodeling causes further occlusion of incompletely coiled aneurysms. Neurosurgery. (2011) 69:598–603. 10.1227/NEU.0b013e3182181c2b 21430583

[3] Chalouhi N Jabbour P Singhal S Drueding R Starke RM Dalyai RT. Stent-assisted coiling of intracranial aneurysms: predictors of complications, recanalization, and outcome in 508 cases. Stroke. (2013) 44:1348–53. 10.1161/STROKEAHA.111.000641 23512976

[4] Frontera JA Moatti J de los Reyes KM McCullough S Moyle H Bederson JB Safety and cost of stent-assisted coiling of unruptured intracranial aneurysms compared with coiling or clipping. J Neurointerv Surg. (2014) 6:65–71. 10.1136/neurintsurg-2012-010544 23223396

[5] Geyik S Yavuz K Yurttutan N Saatci I Cekirge HS. Stent-assisted coiling in endovascular treatment of 500 consecutive cerebral aneurysms with long-term follow-up. AJNR Am J Neuroradiol. (2013) 34:2157–62. 10.3174/ajnr.A3574 23886748 PMC7964833

[6] Lylyk P Miranda C Ceratto R Ferrario A Scrivano E Luna HR. Curative endovascular reconstruction of cerebral aneurysms with the pipeline embolization device: the Buenos Aires experience. Neurosurgery. (2009) 64:632–42. 10.1227/01.NEU.0000339109.98070.65 19349825

[7] Turner RD Turk A Chaudry I. Low-profile visible intraluminal support device: immediate outcome of the first three US cases. J Neurointerv Surg. (2013) 5:157–60. 10.1136/neurintsurg-2011-010187 22345109

[8] Cho YD Sohn CH Kang HS Kim JE Cho WS Hwang G Coil embolization of intracranial saccular aneurysms using the Low-profile Visualized Intraluminal Support (LVIS) device. Neuroradiology. (2014) 56:543–51. 10.1007/s00234-014-1363-x 24740581

[9] Feng Z Fang Y Xu Y Hong B Zhao W Liu J. The safety and efficacy of low profile visualized intraluminal support (LVIS) stents in assisting coil embolization of intracranial saccular aneurysms: a single center experience. J Neurointerv Surg. (2016) 8:1192–6. 10.1136/neurintsurg-2015-012090 26747876

[10] Ge H Lv X Yang X He H Jin H Li Y. LVIS stent versus Enterprise stent for the treatment of unruptured intracranial aneurysms. World Neurosurg. (2016) 91:365–70. 10.1016/j.wneu.2016.04.057 27113398

[11] Zhang X Zhong J Gao H Xu F Bambakidis NC. Endovascular treatment of intracranial aneurysms with the LVIS device: a systematic review. J Neurointerv Surg. (2016) 9:553–7. 10.1136/neurintsurg-2016-012403 27206450

[12] Poncyljusz W Biliński P Safranow K Baron J Zbroszczyk M Jaworski M. The LVIS/LVIS Jr. stents in the treatment of wide-neck intracranial aneurysms: multicentre registry. J Neurointerv Surg. (2015) 7:524–9. 10.1136/neurintsurg-2014-011229 24827067

[13] Fiorella D Arthur A Boulos A Diaz O Jabbour P Pride L. Final results of the US humanitarian device exemption study of the low-profile visualized intraluminal support (LVIS) device. J Neurointerv Surg. (2016) 8:894–7. 10.1136/neurintsurg-2015-011937 26391016

[14] Luo B Yang X Wang S Li H Chen J Yu H. High shear stress and flow velocity in partially occluded aneurysms prone to recanalization. Stroke. (2011) 42:745–53. 10.1161/STROKEAHA.110.593517 21233477

[15] Li C Wang S Chen J Yu H Zhang Y Jiang F. Influence of hemodynamics on recanalization of totally occluded intracranial aneurysms: a patient-specific computational fluid dynamic simulation study. J Neurosurg. (2012) 117:276–83. 10.3171/2012.5.JNS111558 22680247

[16] Sugiyama S Niizuma K Sato K Rashad S Kohama M Endo H. Blood flow into basilar tip aneurysms: a predictor for recanalization after coil embolization. Stroke. (2016) 47:2541–7. 10.1161/STROKEAHA.116.013555 27625377

[17] Yu SC Kwok CK Cheng PW Chan KY Lau SS Lui WM. Intracranial aneurysms: midterm outcome of pipeline embolization device—a prospective study in 143 patients with 178 aneurysms. Radiology. (2012) 265:893–901. 10.1148/radiol.12120422 22996749

[18] Kallmes DF Hanel R Lopes D Boccardi E Bonafé A Cekirge S. International retrospective study of the pipeline embolization device: a multicenter aneurysm treatment study. AJNR Am J Neuroradiol. (2015) 36:108–15. 10.3174/ajnr.A4111 25355814 PMC7965920

[19] Wiebers DO Whisnant JP Huston J Meissner I Brown RD Piepgras DG. Unruptured intracranial aneurysms: natural history, clinical outcome, and risks of surgical and endovascular treatment. Lancet. (2003). 362:103–10. 10.1016/S0140-6736(03)13860-3 12867109

[20] Roy D Milot G Raymond J. Endovascular treatment of unruptured aneurysms. Stroke. (2001) 32:1998–2004. 10.1161/hs0901.095600 11546888

[21] Paliwal N Yu H Xu J Xiang J Siddiqui A Yang X. Virtual stenting workflow with vessel-specific initialization and adaptive expansion for neurovascular stents and flow diverters. Comput Methods Biomech Biomed Eng. (2016) 19:1423–31. 10.1080/10255842.2016.1149573 26899135 PMC4945427

[22] Wang C Tian Z Liu J Jing L Paliwal N Wang S. Flow diverter effect of LVIS stent on cerebral aneurysm hemodynamics: a comparison with Enterprise stents and the Pipeline device. J Transl Med. (2016) 14:199. 10.1186/s12967-016-0959-9 27370946 PMC4930570

[23] Mitsos AP Kakalis NM Ventikos YP Byrne JV. Haemodynamic simulation of aneurysm coiling in an anatomically accurate computational fluid dynamics model: technical note. Neuroradiology. (2008) 50:341–7. 10.1007/s00234-007-0334-x 18043912

[24] Tremmel M Xiang J Natarajan SK Hopkins LN Siddiqui AH Levy EI. Alteration of intra-aneurysmal hemodynamics for flow diversion using enterprise and vision stents. World Neurosurg. (2010) 74:306–15. 10.1016/j.wneu.2010.05.008 21197155 PMC3011938

[25] Levitt MR McGah PM Aliseda A Mourad PD Nerva JD Vaidya SS. Cerebral aneurysms treated with flow-diverting stents: computational models with intravascular blood flow measurements. AJNR Am J Neuroradiol. (2014) 35:143–8. 10.3174/ajnr.A3624 23868162 PMC3858573

[26] Kojima M Irie K Fukuda T Arai F Hirose Y Negoro M. The study of flow diversion effects on aneurysm using multiple enterprise stents and two flow diverters. Asian J Neurosurg. (2012) 7:159–65. 10.4103/1793-5482.106643 23559981 PMC3613636

[27] Liu J Jing L Wang C Paliwal N Wang S Zhang Y. Effect of hemodynamics on outcome of subtotally occluded paraclinoid aneurysms after stent-assisted coil embolization. J Neurointerv Surg. (2016) 8:1140–7. 10.1136/neurintsurg-2015-012050 26610731 PMC4882272

[28] Zhang Q Jing L Liu J Wang K Zhang Y Paliwal N. Predisposing factors for recanalization of cerebral aneurysms after endovascular embolization: a multivariate study. J Neurointerv Surg. (2017) 10:252–7. 10.1136/neurintsurg-2017-013041 28377443 PMC5826759

[29] Murias Quintana E Gil Garcia A Vega Valdés P Cuellar H Meilán Martínez Á Saiz Ayala A. Anatomical results, rebleeding and factors that affect the degree of occlusion in ruptured cerebral aneurysms after endovascular therapy. J Neurointerv Surg. (2015) 7:892–7. 10.1136/neurintsurg-2014-011300 25358516

[30] Fujimura S Takao H Suzuki T Dahmani C Ishibashi T Mamori H. Hemodynamics and coil distribution with changing coil stiffness and length in intracranial aneurysms. J Neurointerv Surg. (2018) 10:797–801. 10.1136/neurintsurg-2017-013457 29259122 PMC6204941

[31] Schirmer CM Malek AM. Critical influence of framing coil orientation on intra-aneurysmal and neck region hemodynamics in a sidewall aneurysm model. Neurosurgery. (2010) 67:1692–702. 10.1227/NEU.0b013e3181f9a93b 21107200

[32] Morales HG Kim M Vivas EE Villa-Uriol MC Larrabide I Sola T. How do coil configuration and packing density influence intra-aneurysmal hemodynamics. AJNR Am J Neuroradiol. (2011) 32:1935–41. 10.3174/ajnr.A2635 21885712 PMC7965998

[33] Sluzewski M van Rooij WJ Slob MJ Bescós JO Slump CH Wijnalda D. Relation between aneurysm volume, packing, and compaction in 145 cerebral aneurysms treated with coils. Radiology. (2004) 231:653–8. 10.1148/radiol.2313030460 15118115

